# Supplementary material for: Aberrant bowel movement frequencies coincide with increased microbe-derived blood metabolites associated with reduced organ function
Source: Cell Rep Med. 2024 Jul 16;5(7):101646. doi: 10.1016/j.xcrm.2024.101646 (PMC11293344; doi:10.1016/j.xcrm.2024.101646)
Supplement: Document S1. Figures S1–S6 and Table S1 [file mmc1.pdf]

Cell Reports Medicine, Volume *5*

Supplemental information

Aberrant bowel movement frequencies coincide with increased microbe-derived blood metabolites associated with reduced organ function

Johannes P. Johnson-Martínez, Christian Diener, Anne E. Levine, Tomasz Wilmanski, David L. Suskind, Alexandra Ralevski, Jennifer Hadlock, Andrew T. Magis, Leroy Hood, Noa Rappaport, and Sean M. Gibbons

SUPPLEMENTAL FIGURES AND TABLES

Covariates:	Mean ± standard deviation, or % across Arivale:
Gender	65.1% Female
BMI	27.2 ± 5.89
Age	46.36 ± 12.96
eGFR	89.07 ± 20.20
CRP	2.40 ± 4.76
LDL	114.17 ± 33.77
A1C	5.49 ± 0.57
Highlighted exclusionary criteria:	
Percent with self-reported kidney disease:	3.00% (119 out of 3,955 participants with BMF data available withheld from cohort)
Percent IBS or IBD:	3.23% (128 out of 3,955 participants with BMF data available withheld from cohort)
Exclusionary features (988 out of 3,955 participants with BMF data, or 25% of the initial BMF cohort, answered affirmatively to any of these and were excluded from the analyses. The final N of remaining participants after merging with covariates was N = 1,425 for the final baseline cohort):	
Self - current history - bladder infection	
Self - current history - kidney disease	
Self - current history - kidney infection	
Self - current history - kidney stones	
Self - current history - bladder/kidney - other	
Self - current history - polycystic kidney disease (PKD)	
Self - current history - urinary incontinence	
Self - current history - kidney cancer	
Self - current history - celiac disease	
Self - current history - colonic Crohn’s disease	
Self - current history - diverticulosis	
Self - current history - gastroesophageal reflux disease (GERD)	
Self - current history - ileal Crohn’s disease	
Self - current history - irritable bowel syndrome (IBS)	
Self - current history - inflammatory bowel disease (IBD)	
Self - current history - ulcerative colitis	
Self - current history - peptic ulcer	
Self - laxatives usage	
Self - anticoagulation or cholesterol drugs usage	
Self - blood pressure drugs usage	

Table S1. The modeling covariates and exclusionary criteria, related to results in Figures 2-7. Out of the 3,955 total Arivale participants that had BMF data, 3.00% self-reported kidney disease (the kidney-related questions in the exclusionary features) and 3.23% self-reported IBS or IBD. An initial baseline cohort of 3,132 participants that had health history survey questionnaire data was available. The participants that answered affirmatively to the exclusionary features were removed from the analysis, resulting in 25% of the initial cohort with BMF data being filtered down to N = 1,561, and subsequently, a final baseline cohort of 1,425 individuals after merging for covariates.

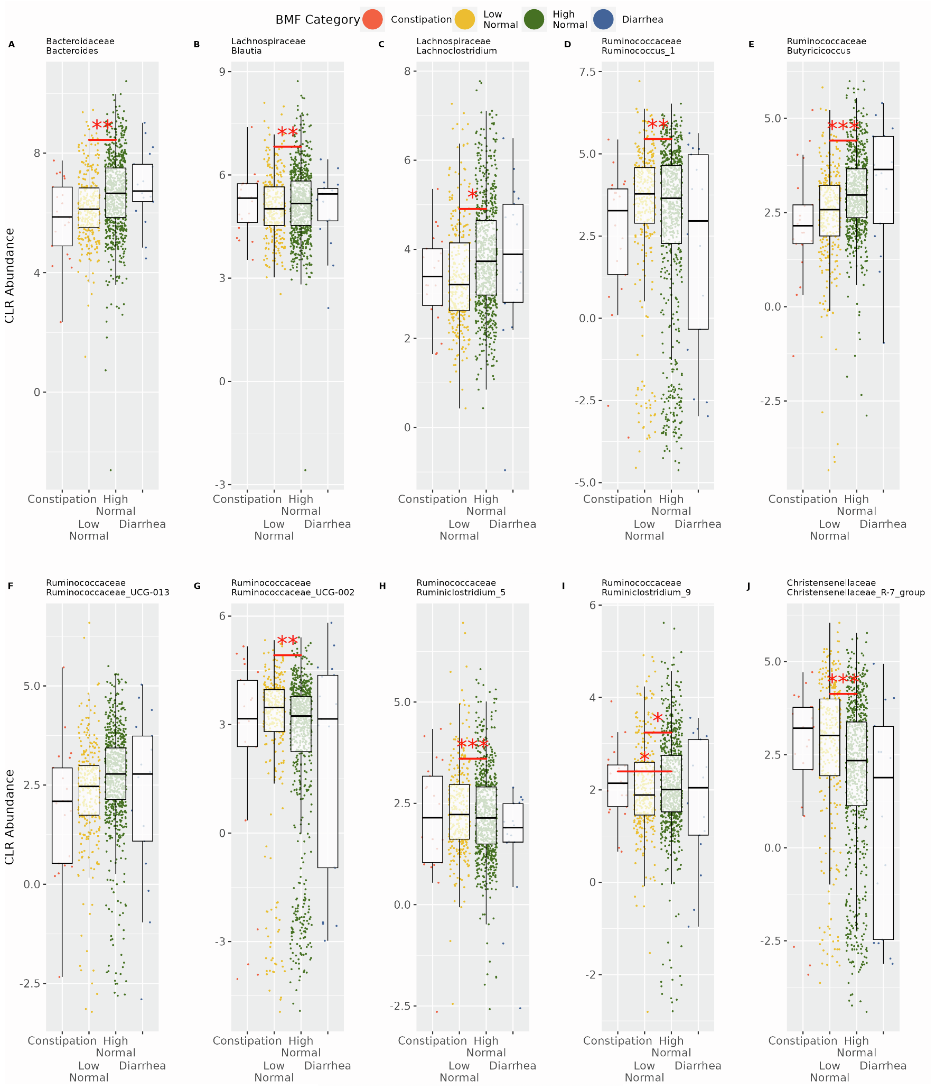

12

Low BMF ↔ High BMF

Figure S1. The top 10 most abundant genera significantly associated with BMF (A-J), related to Figure 4. Significant genera from the CORNCOB analysis in order of decreasing CLR-transformed abundance. The line in each plot denotes significant differences from the reference category (“High Normal” BMF), and asterisks denote FDR-corrected significance threshold. (***): $p < 0.0001$, (**): $0.0001 < p < 0.01$, (*): $0.01 < p < 0.05$. The horizontal axes are annotated as four BMF categories: “Constipation” (BMF = 1-2× per week), “Low Normal” (BMF = 3-6× per week), “High Normal” (BMF = 1-3× per day) which is the reference category in regression, and “Diarrhea” (BMF = 4× or more per day).

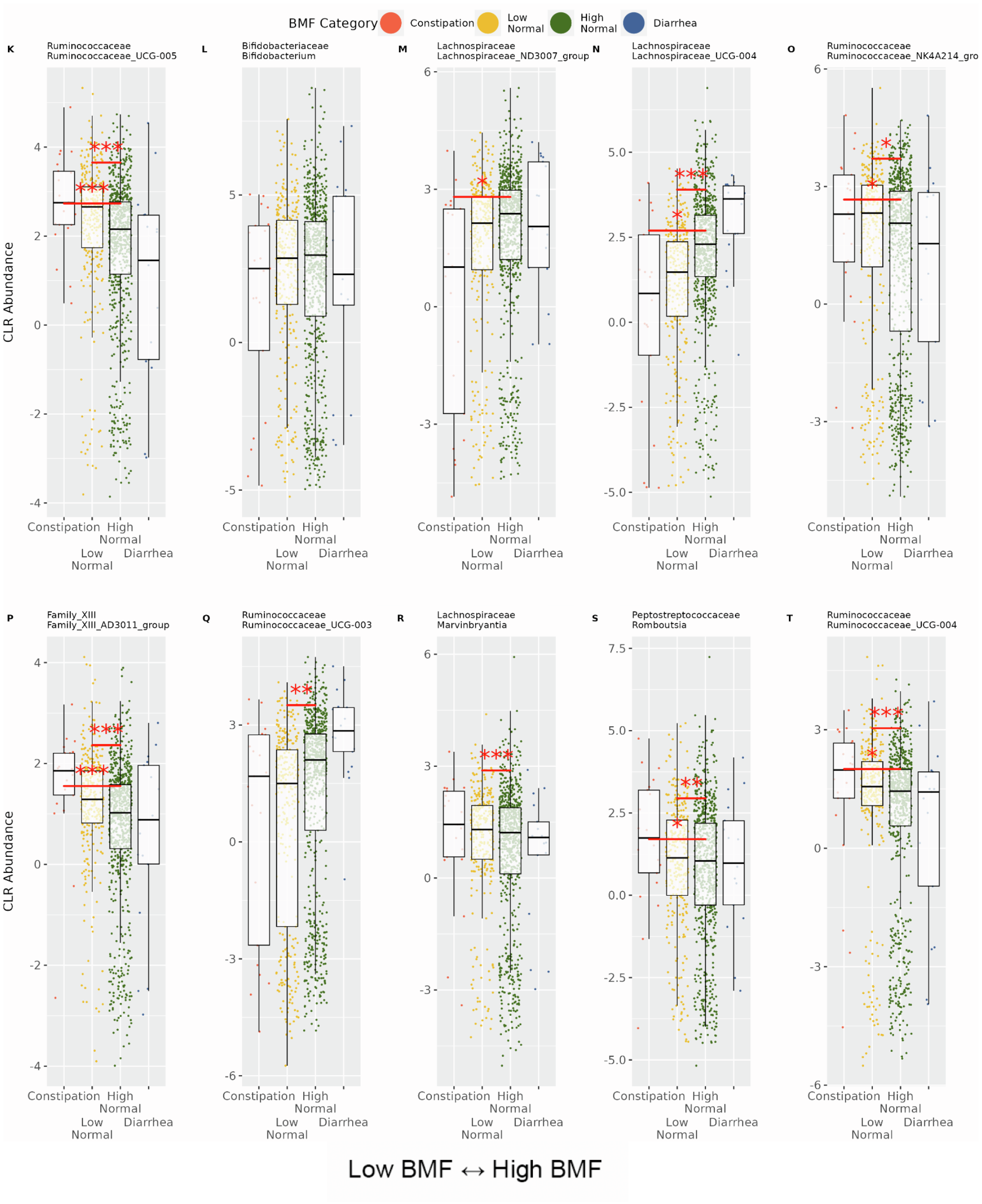
BMF Category
Constipation
Low Normal
High Normal
Diarrhea
K Ruminococcaceae Ruminococcaceae_UCG-005
L Bifidobacteriaceae Bifidobacterium
M Lachnospiraceae Lachnospiraceae_ND3007_group
N Lachnospiraceae Lachnospiraceae_UCG-004
O Ruminococcaceae Ruminococcaceae_NK4A214_gro
P Family_XIII Family_XIII_AD3011_group
Q Ruminococcaceae Ruminococcaceae_UCG-003
R Lachnospiraceae Marvinbryantia
S Peptostreptococcaceae Romboutsia
T Ruminococcaceae Ruminococcaceae_UCG-004
CLR Abundance
Constipation
Low Normal
High Normal
Diarrhea
Low BMF ↔ High BMF

Figure S2. The top 11-20 most abundant genera associated with BMF (K-T), related to Figure 4. Significant genera from the CORNCOB analysis in order of decreasing CLR-transformed abundance. The line in each plot denotes significant differences from the reference category (“High Normal” BMF), and asterisks denote FDR-corrected significance threshold. (***): $p < 0.0001$, (**): $0.0001 < p < 0.01$, (*): $0.01 < p < 0.05$. The horizontal axes are annotated as four BMF categories: “Constipation” (BMF = 1-2× per week), “Low Normal” (BMF = 3-6× per week), “High Normal” (BMF = 1-3× per day) which is the reference category in regression, and “Diarrhea” (BMF = 4× or more per day).

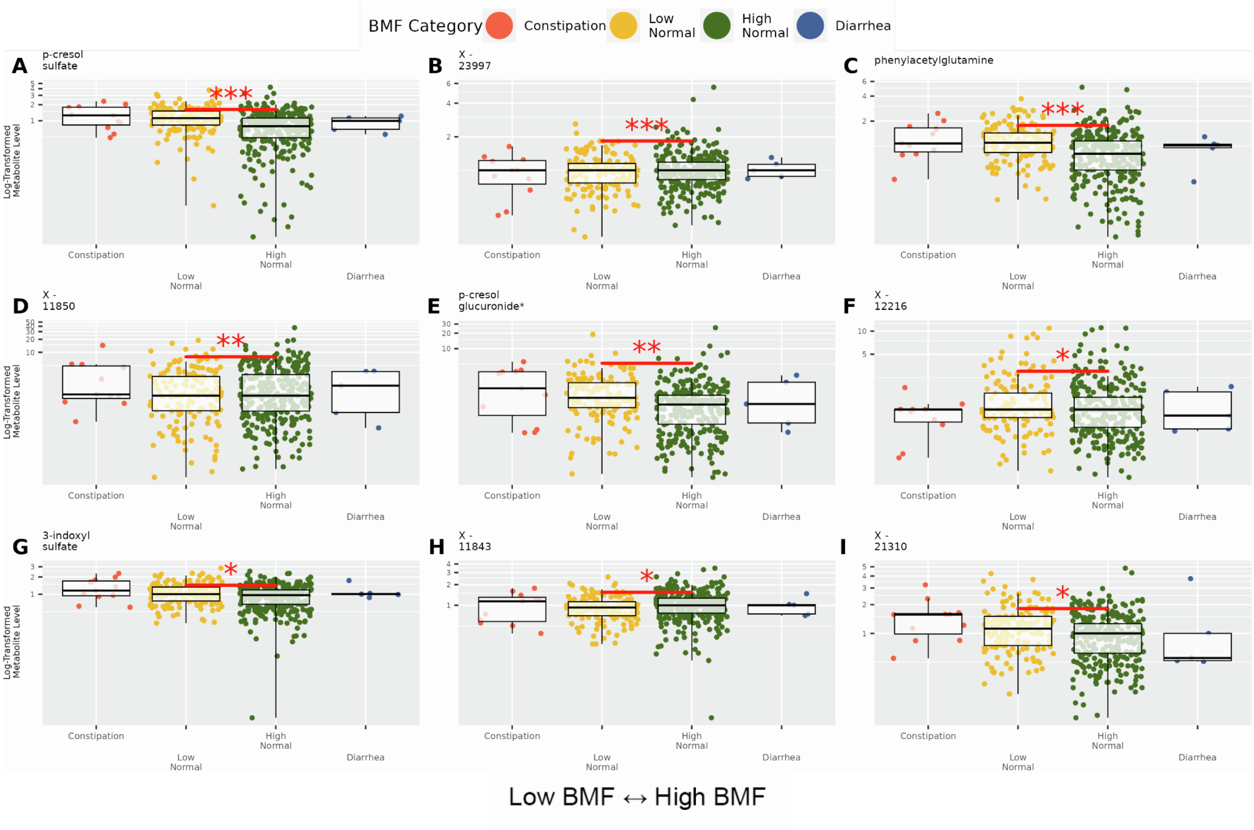

Figure S3. Significant BMF-associated plasma metabolites boxplots (A-I), related to Figure 5. Significant plasma metabolites from the LIMMA analysis. The horizontal axes are annotated as four BMF categories: “Constipation” (BMF = 1-2✕ per week), “Low Normal” (BMF = 3-6✕ per week), “High Normal” (BMF = 1-3✕ per day) which is the reference category in regression, and “Diarrhea” (BMF = 4✕ or more per day). Red significant comparison lines across each plot denote significant differences from the reference category (“High Normal” BMF), and asterisks denote FDR-corrected significance threshold. (***): $p < 0.0001$, (**): $0.0001 < p < 0.01$, (*): $0.01 < p < 0.05$.

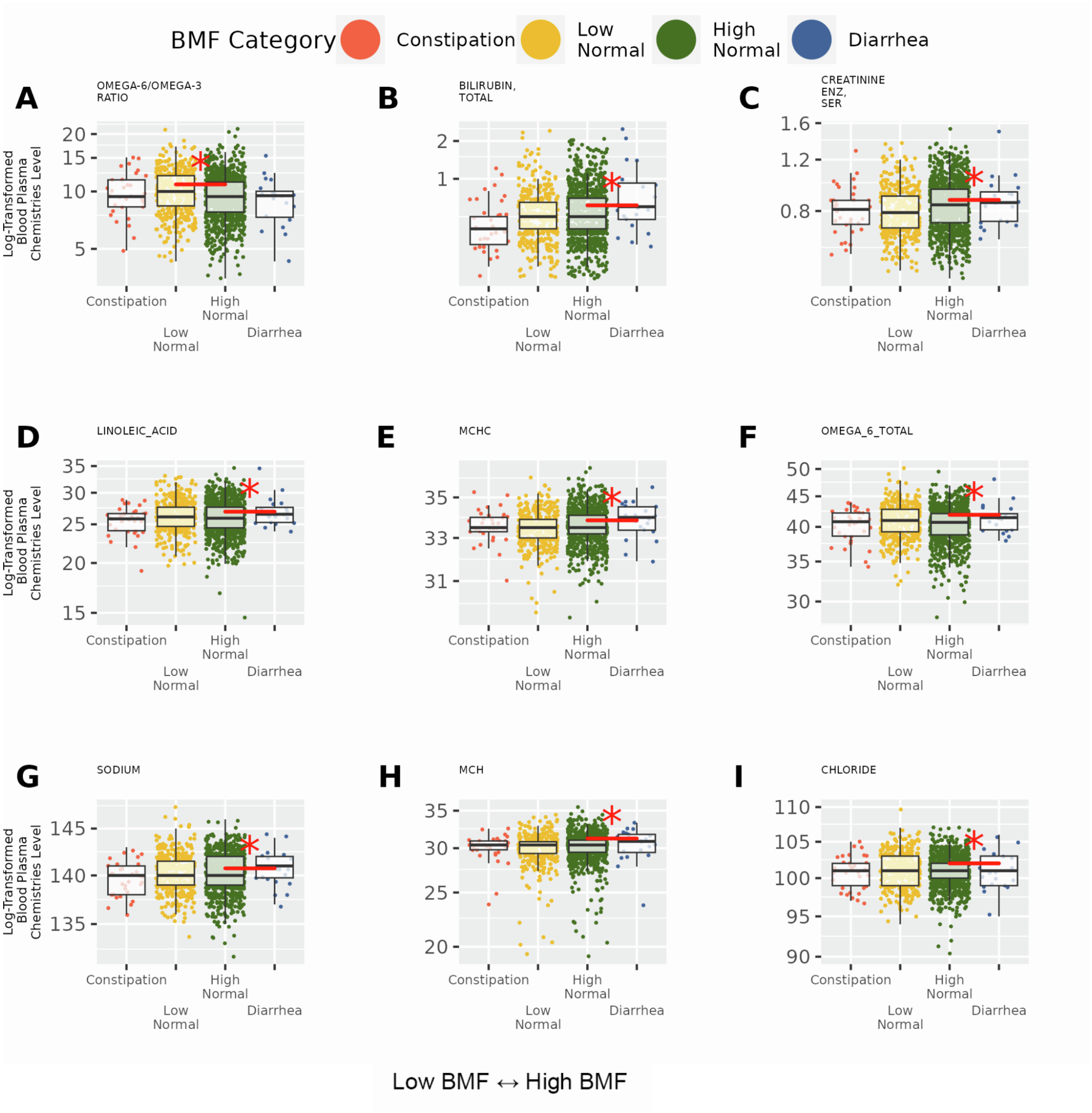

BMF Category
Constipation
Low Normal
High Normal
Diarrhea
A
OMEGA-6/OMEGA-3 RATIO
B
BILIRUBIN, TOTAL
C
CREATININE ENZ, SER
D
LINOLEIC_ACID
E
MCHC
F
OMEGA_6_TOTAL
G
SODIUM
H
MCH
I
CHLORIDE
Log-Transformed Blood Plasma Chemistries Level
Low BMF ↔ High BMF

Figure S4. Significant BMF-associated clinical chemistries boxplots (A-I), related to Figure 6. Significant clinical chemistries from the LIMMA analysis. The horizontal axes are annotated as four BMF categories: “Constipation” (BMF = 1-2× per week), “Low Normal” (BMF = 3-6× per week), “High Normal” (BMF = 1-3× per day) which is the reference category in regression, and “Diarrhea” (BMF = 4× or more per day). Red significant comparison lines across each plot denote significant differences from the reference category (“High Normal” BMF), and asterisks denote FDR-corrected significance threshold. (***): $p < 0.0001$, (**): $0.0001 < p < 0.01$, (*): $0.01 < p < 0.05$.

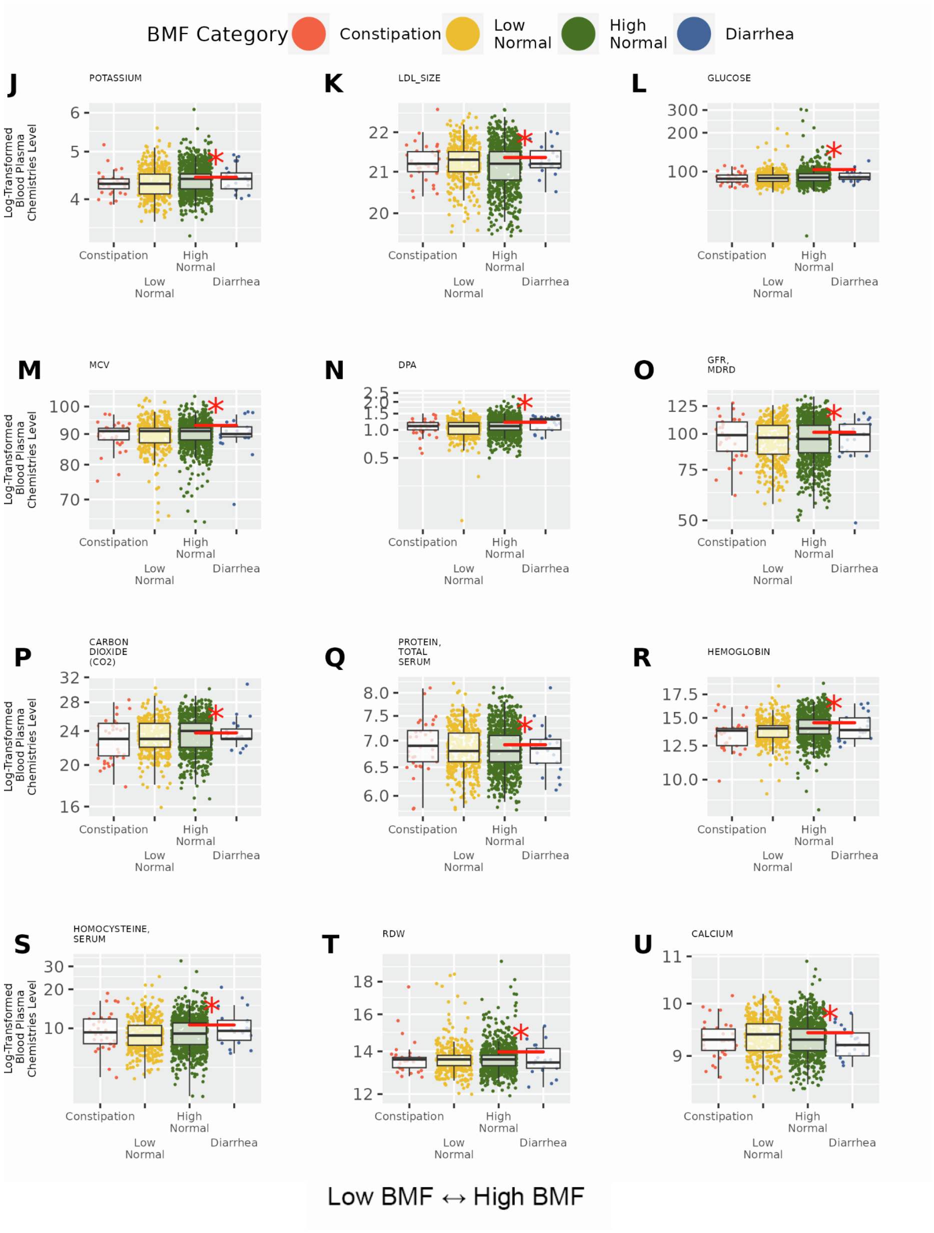

BMF Category
Constipation
Low Normal
High Normal
Diarrhea
J
POTASSIUM
K
LDL_SIZE
L
GLUCOSE
M
MCV
N
DPA
O
GFR, MDRD
P
CARBON DIOXIDE (CO2)
Q
PROTEIN, TOTAL SERUM
R
HEMOGLOBIN
S
HOMOCYSTEINE, SERUM
T
RDW
U
CALCIUM
Log-Transformed Blood Plasma Chemistries Level
Low BMF ↔ High BMF

Figure S5. The remaining significant BMF-associated clinical chemistries boxplots (J-U), related to Figure 6. The remaining significant clinical chemistries from the LIMMA analysis. The horizontal axes are annotated as four BMF categories: "Constipation" (BMF = 1-2✕ per week), "Low Normal" (BMF = 3-6✕ per week), "High Normal" (BMF = 1-3✕ per day) which is the reference category in regression, and "Diarrhea" (BMF = 4✕ or more per day). Red significant comparison lines across each plot denote significant differences from the reference category ("High Normal" BMF), and asterisks denote FDR-corrected significance threshold. (***): $p < 0.0001$, (**): $0.0001 < p < 0.01$, (*): $0.01 < p < 0.05$.

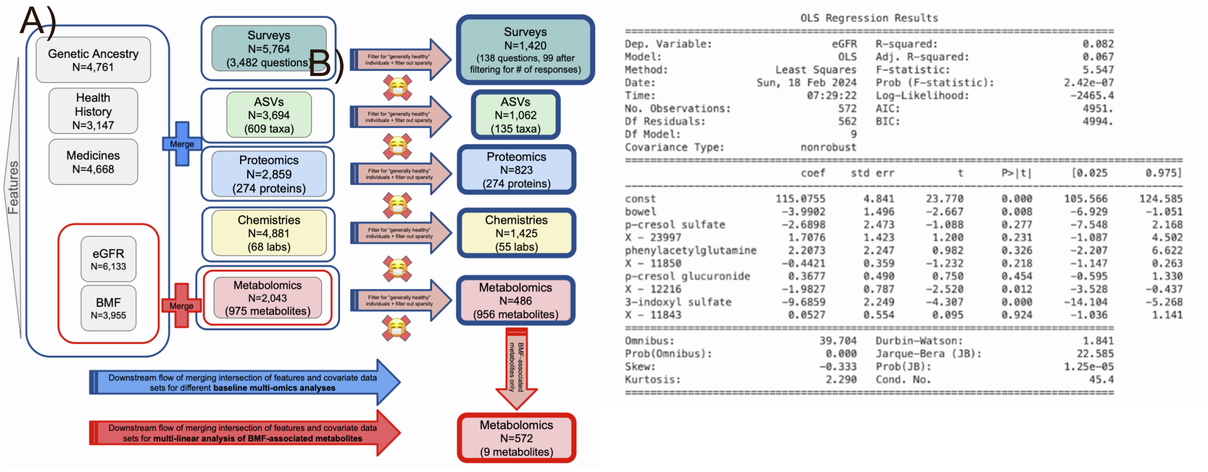

Figure S6. A) Flow Chart for Cohort Selection of Baseline Population, related to the Generally-healthy cohort section of the STAR Methods. B) OLS regression resulting from eGFR ~ BMF-associated metabolites + BMF, related to Figure 7B. A) Individuals with the full complement of covariate data (gender, age, BMI, and CRP, LDL, A1C, and PCs 1-3) were further filtered for having available baseline data for each of the following: surveys, microbiome profiles, proteomics, clinical chemistries (e.g. complete blood count, or CBC; and comprehensive metabolic panel, or CMP) and metabolomics. The “generally-healthy” exclusion criteria were then imposed (38.5% excluded; see Method Details), along with sparsity or non-missingness minimums for the features in the ‘omics data (≥ 30% prevalence for gut microbiome data, metabolomics and clinical chemistries; ≥ 50% prevalence for proteomics; and ≥ 90% prevalence and ≥ 10% affirmative for binary responses in the survey questions). These filters resulted in the final sub-cohort numbers shown on the right side of the figure in blue outlines. Additionally, the eGFR and BMF data frames were merged with the metabolomics data frame and filtered by the “generally-healthy” exclusionary criteria for the 9 BMF-associated metabolites eGFR regression and mediation analysis. B) The p-value for the overall generalized-linear model (eGFR ~ BMF-related metabolites) was significant (N = 572, p = 2.42E-7, R^2 = 0.082) and so were the p-values of the individual β-coefficients for 3-IS ($\beta_{3\text{-IS}}$ = -9.69, p = 1.96E-5), BMF (denoted “bowel”; β_{BMF} = -3.99, p = 7.88E-3), and X - 12216 ($\beta_{X-12216}$ = -1.98, p = 1.20E-2).
